# Heart-rate and rating-of-perceived-exertion running-speed thresholds show acceptable test-retest reliability

**DOI:** 10.1080/15502783.2025.2550178

**Published:** 2025-08-22

**Authors:** Logan Sallas, Brandi Antonio, David H. Fukuda, Jeffrey R. Stout

**Affiliations:** University of Central Florida, Physiology of Work and Exercise Response (POWER) Lab, Institute of Exercise Physiology and Rehabilitation Science, Orlando, FL, USA

**Keywords:** GXT, heart rate, running, threshold

## Abstract

**Purpose:**

This study examined the test-retest reliability of heart-rate- (HR) and rating-of-perceived-exertion- (RPE) derived running-speed thresholds (RS_thr) in trained men.

**Methods:**

Fourteen healthy, aerobically trained men (20.6 ± 1.9 y; VO₂peak 50.5 ± 7.8 mL·kg^−1^ ·min^−1^) performed a graded-exercise test (GXT) to determine VO₂peak and maximum running speed (MRS). After a 5-min warm-up, the GXT began at 8 km·h^−1^ (0% grade) and increased by 1 km·h^−1^ every 3 min until volitional exhaustion. Participants then completed two visits with four 8-min trials at 60%, 70%, 80%, and 90% MRS in randomized order. Trials were separated by ≤15 min or until HR returned to within 10% of post-warm-up. HR was recorded continuously; RPE (Borg 6–20) was sampled every 30s. The rate of rise (Δvariable/Δt) for each trial was regressed against speed, and the y-intercept defined RS_thr (RS_HR, RS_RPE). Reliability statistics included ICC₂,₁, coefficient of variation (CV %), standard error of measurement (SEM), and minimum detectable change at 95% confidence (MDC₉₅).

**Results:**

Twelve RS_HR and fourteen RS_RPE data sets met quality criteria. RS values with a z-score ≥2 were identified as potential outliers and excluded from the final analysis. RS_HR demonstrated good reliability (ICC = 0.86, 95% CI 0.32–0.96; SEM = 0.73 km/hr; CV = 7.0%; MDC₉₅ = 2.01 km/hr). RS_RPE demonstrated moderate reliability (ICC = 0.75, 95% CI 0.17–0.89; SEM = 1.08 km/hr; CV = 11.96%; MDC₉₅ = 3.11 km/hr).

**Conclusion:**

Eight-minute submaximal trials yield HR- and RPE-derived running-speed thresholds with acceptable repeatability in trained young men. HR provides tighter error bands, yet RPE remains a practical alternative when instrumentation is unavailable. Future work should include women, untrained individuals, and validation against physiological thresholds to enhance generalizability.

